# QiShenYiQi Inhibits Tissue Plasminogen Activator–Induced Brain Edema and Hemorrhage after Ischemic Stroke in Mice

**DOI:** 10.3389/fphar.2021.759027

**Published:** 2022-01-12

**Authors:** Yang Ye, Quan Li, Chun-Shui Pan, Li Yan, Kai Sun, Xiao-Yi Wang, Shu-Qi Yao, Jing-Yu Fan, Jing-Yan Han

**Affiliations:** ^1^ Department of Integration of Chinese and Western Medicine, School of Basic Medical Sciences, Peking University, Beijing, China; ^2^ Tasly Microcirculation Research Center, Peking University Health Science Center, Beijing, China; ^3^ Academy of Integration of Chinese and Western Medicine, Peking University Health Science Center, Beijing, China; ^4^ Key Laboratory of Microcirculation, State Administration of Traditional Chinese Medicine of the People’s Republic of China, Beijing, China; ^5^ Key Laboratory of Stasis and Phlegm, State Administration of Traditional Chinese Medicine of the People’s Republic of China, Beijing, China; ^6^ State Key Laboratory of Core Technology in Innovative Chinese Medicine, Tianjin, China

**Keywords:** blood-brain barrier, hemorrhage, ischemic stroke, QiShenYiQi, tissue plasminogen activator

## Abstract

**Background:** Thrombolysis with tissue plasminogen activator (tPA) remains the only approved drug therapy for acute ischemic stroke. However, delayed tPA treatment is associated with an increased risk of brain hemorrhage. In this study, we assessed whether QiShenYiQi (QSYQ), a compound Chinese medicine, can attenuate tPA-induced brain edema and hemorrhage in an experimental stroke model.

**Methods:** Male mice were subjected to ferric chloride-induced carotid artery thrombosis followed by mechanical detachment of thrombi. Then mice were treated with QSYQ at 2.5 h followed by administration of tPA (10 mg/kg) at 4.5 h. Hemorrhage, infarct size, neurological score, cerebral blood flow, Evans blue extravasation, FITC-labeled albumin leakage, tight and adherens junction proteins expression, basement membrane proteins expression, matrix metalloproteinases (MMPs) expression, leukocyte adhesion, and leukocyte infiltration were assessed 24 h after tPA administration.

**Results:** Compared with tPA alone treatments, the combination therapy of QSYQ and tPA significantly reduced hemorrhage, infarction, brain edema, Evans blue extravasation, albumin leakage, leukocyte adhesion, MMP-9 expression, and leukocyte infiltration at 28.5 h after stroke. The combination also significantly improved the survival rate, cerebral blood flow, tight and adherens junction proteins (occludin, claudin-5, junctional adhesion molecule-1, zonula occludens-1, VE-cadherin, α-catenin, β-catenin) expression, and basement membrane proteins (collagen IV, laminin) expression. Addition of QSYQ protected the downregulated ATP 5D and upregulated p-Src and Caveolin-1 after tPA treatment.

**Conclusion:** Our results show that QSYQ inhibits tPA-induced brain edema and hemorrhage by protecting the blood-brain barrier integrity, which was partly attributable to restoration of energy metabolism, protection of inflammation and Src/Caveolin signaling activation. The present study supports QSYQ as an effective adjunctive therapy to increase the safety of delayed tPA thrombolysis for ischemic stroke.

## Introduction

Intravenous thrombolysis with tissue plasminogen activator (tPA) is the only US Food and Drug Administration-approved drug therapy for acute ischemic stroke (Zhang et al., 2020). However, tPA has to be administered within 3 h and selected patients would be increased to 4.5 h after symptom onset (De Keyser et al., 2007; Cheng and Kim, 2015). Delayed tPA treatment may be related to increased risks of brain edema and hemorrhage (Mao et al., 2017). Therefore, potential therapies that decrease the risks of brain edema and hemorrhage caused by delayed tPA administration are greatly needed.

Blood-brain barrier (BBB) disruption plays a critical role in the pathogenesis of tPA-associated brain edema and hemorrhage (Ye et al., 2020). The tight junctions (TJ) and adherens junctions (AJ) of endothelial cells, and cerebrovascular basement membrane are important components of BBB (Zhao et al., 2015). After ischemic stroke, delayed tPA treatment directly induces degradation of TJ, AJ, and basement membrane due to the protease activity of tPA and plasmin (Niego and Medcalf, 2014; Chen QF. et al., 2018). The neurotoxic effects of tPA have been reported to aggravate the pathological process of stroke (Harada et al., 2005). Many researches also indicated that tPA increases matrix metalloproteinase-9 (MMP-9) activity in the ischemic brain, which exacerbates BBB disruption by degrading TJ, AJ, and basement membrane (Jin et al., 2011; Ding et al., 2019). Besides, tPA-induced reperfusion injury after thrombolysis causes further damage of BBB (Knecht et al., 2017; Chen QF. et al., 2018). The complex mechanism behind tPA-associated BBB disruption makes it hard to attenuate edema and hemorrhage through targeting a single link.

QiShenYiQi (QSYQ) is a compound Chinese medicine approved by the China Food and Drug Administration in 2003 for treatment of myocardial ischemia (Zheng et al., 2019). It is composed of four Chinese herbs, i.e., *Astragalus membranaceus* (Huangqi), *Salvia miltiorrhiza* (Danshen), *Panax notoginseng* (Sanqi), and *Dalbergia odorifera* (Jiangxiang). QSYQ and its major bioactive ingredients exert cardioprotection against myocardial ischemia-reperfusion (IR) injury via multiple mechanisms (Han et al., 2019). Several bioactive ingredients of QSYQ have presented potential to protect the brain from IR injury (Hou et al., 2016; Wang et al., 2017; Xie et al., 2018). Our previous study has demonstrated that T541, a compound medicine consisting of three major bioactive ingredients of QSYQ (total astragalus saponins, total salvianolic acids, and total panax notoginseng saponins), attenuates delayed tPA-related angioedema and hemorrhage by preventing the disruption of BBB (Chen QF. et al., 2018). Recent studies showed that QSYQ exerts protective effects against acute cerebral IR injury via inhibiting neuroinflammatory response (Wang et al., 2020; Wang et al., 2021). The available evidence indicates that QSYQ is potentially able to protect BBB integrity and inhibit tPA-associated edema and hemorrhage after stroke. In the present study, we aimed to determine whether and how QSYQ attenuates tPA-induced brain edema and hemorrhage in a mouse model of ischemic stroke.

## Materials and Methods

### Animal Model

All animal protocols were approved by the Committee on the Ethics of Animal Experiments of Peking University Health Science Center. Male C57BL/6N mice (six to seven weeks of age) weighing 20–24 g were purchased from Beijing Vital River Laboratory (Certificate No. SCXK 2016-0006, Beijing, China). The cerebral thrombus model was established by ferric chloride (FeCl_3_) stimulation of the carotid artery followed by mechanical detachment of thrombus as described (Martinez de Lizarrondo et al., 2017). Briefly, a mouse was anesthetized with sodium pentobarbital (2%, 45 mg/kg, I.P.) and placed under a dissecting microscope. The right common carotid artery was exposed and wrapped with a piece of filter paper strip soaked in 20% FeCl_3_ solution (Fuchen Chemicals, Tianjin, China) for 5 min. The filter paper was then removed to allow the formation of a thrombus for 10 min. Thereafter, the thrombus was detached by a microforcep and migrated toward the intracranial circulation. Cerebral blood flow (CBF) was measured 2.5 h after surgery, and only mice with percent CBF at 2.5 h falling by 60–90% compared to contralateral sides were used for further experiments (Supplementary Figure S1). Mice in the sham group underwent the same operation but the FeCl_3_ solution was replaced with saline.

### Experimental Design

**FIGURE 1:**
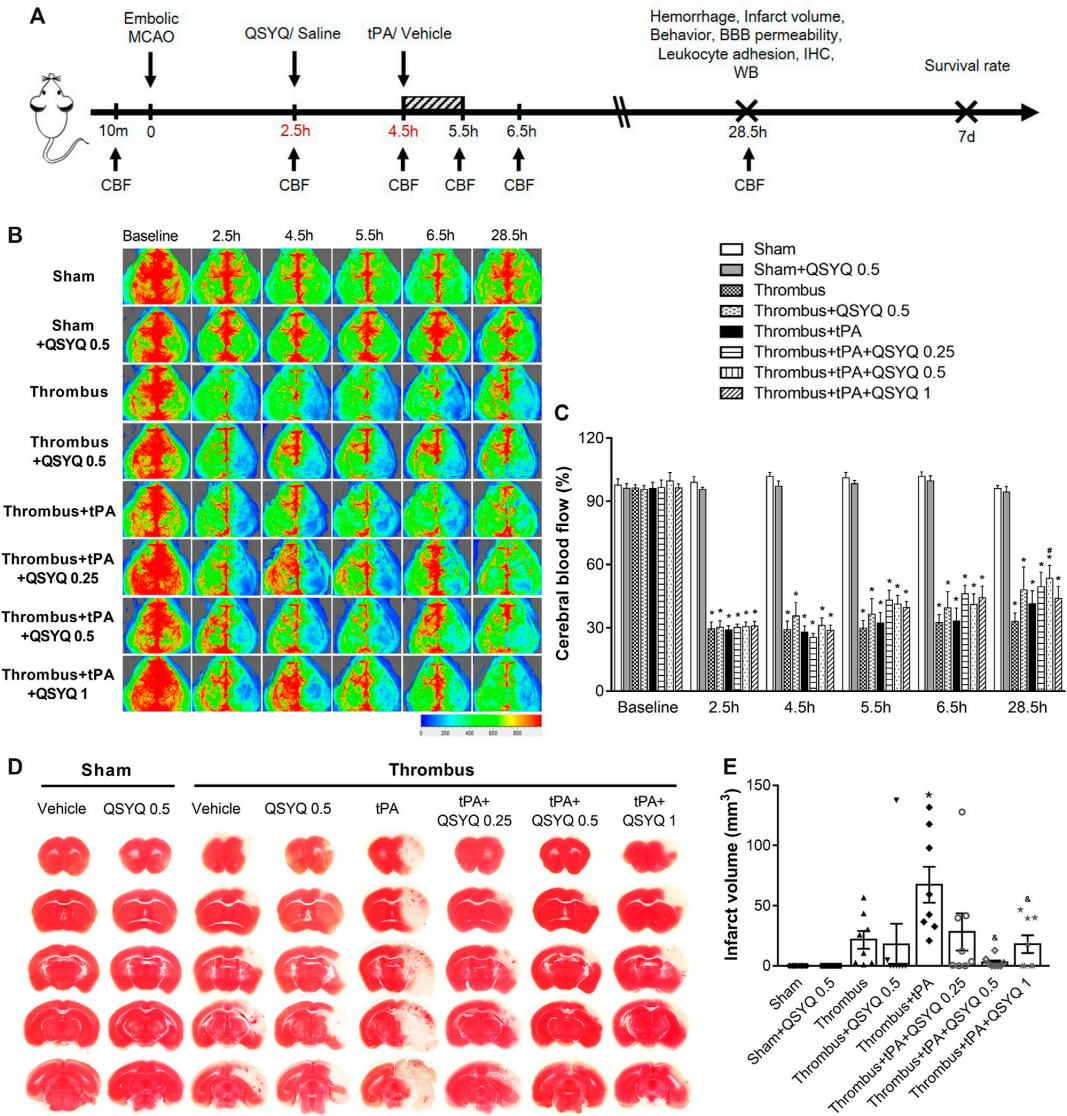
Effect of QSYQ on cerebral blood flow (CBF) and infarction in tPA-treated stroke mice. **(A)** Schematic representation of experimental design. **(B)** Representative images and **(C)** quantitative analysis of CBF at baseline, 2.5, 4.5, 5.5, 6.5, and 28.5 h after stroke onset (n = six to seven per group). Data were compared by a two-way ANOVA followed by Tukey’s post hoc test. **(D)** Representative images and **(E)** quantitative analysis of infarct volume (n = 8 per group). Data were compared by a two-way ANOVA followed by Tukey’s post hoc test. CBF indicates cerebral blood flow; MCAO, middle cerebral artery occlusion; BBB, blood-brain barrier; IHC, immunohistochemistry; WB, western blot. *p < 0.05 vs sham; ^**#**^ *p* < 0.05 vs thrombus; ^&^ *p* < 0.05 vs thrombus + tPA.

Mice were randomly assigned to eight groups via random number: 1) Sham + Vehicle, 2) Sham + QSYQ 0.5, 3) Thrombus + Vehicle, 4) Thrombus + QSYQ 0.5, 5) Thrombus + tPA, 6) Thrombus + tPA + QSYQ 0.25, 7) Thrombus + tPA + QSYQ 0.5, and 8) Thrombus + tPA + QSYQ 1 (Supplementary Figure S2). In QSYQ groups, the animals were given QSYQ at 2.5 h after surgery (Shao et al., 2017) through a gavage tube at 0.25 g/kg, 0.5 g/kg, or 1 g/kg, respectively. The dose of 0.25 g/kg is equivalent to clinical dose of QSYQ for adult (Shang et al., 2013). QiShenYiQi were obtained from Tasly Pharmaceutical Co. Ltd. (Batch No. 190308, Tianjin, China), and dissolved in 0.2 ml saline. The animals in vehicle groups were given equal volume of saline the same way. The animals in tPA groups were administrated with tPA (Actilyse, Boehringer Ingelheim, Biberach, Gemany) at 10 mg/kg by continuous infusion through left femoral vein for an hour starting from 4.5 h after ischemia onset (Figure 1A). The dose of tPA was determined according to previous reports (Mao et al., 2017). Only the optimal dosage of QSYQ was used in further experiments. The number of mice used in each experiment is summarized in Supplementary Table S1.

### Measurement of Infarction and Cerebral Water Content

Mice were euthanatized and transcardially perfused with saline 24 h after tPA administration. The brains were removed quickly and were sliced into five serial 2-mm-thick coronal sections. Brain slices were immersed in 2,3,5-triphenyltetrazolium chloride (2%, Sigma-Aldrich, MO, United States) for 20 min. The slices were photographed to quantify infarct volume. For assessment of cerebral water content, mice were sacrificed at 24 h after tPA treatment. Brains were quickly removed and weighted as wet weight. Then the brains were placed in a 60°C oven for 3 days and the dry weight was recorded. Cerebral water content was calculated as per (wet weight-dry weight)/wet weight×100%.

### Assessment of Intracerebral Hemorrhage

Mice were perfused with saline transcardially 24 h after administration of tPA. Brains were removed immediately and sliced into five serial slices. The slices were photographed followed by homogenization and centrifugation. Hemoglobin content was measured using a hemoglobin colorimetric assay kit (Item No. 700540, Cayman Chemical, MI, United States). The severity of brain hemorrhage was classified as four types following previously described criteria: no hemorrhage (NH); hemorrhagic infarction type 1 (HI-1), revealing a single petechia in the ischemic area; hemorrhagic infarction type 2 (HI-2), two to three petechiae in the ischemic area; and hemorrhagic infarction type 3 (HI-3), more than three petechiae in the ischemic area (Garcia-Culebras et al., 2017). The hemorrhage rate was calculated as (1-number of NH/overall number of mice)×100%.

### Evaluation of BBB Permeability

Mice were intravenously injected with Evans blue (2%, 4 ml/kg, Sigma-Aldrich, MO, United States) 3 h before they were sacrificed to assess the BBB permeability. Mice were transcardially perfused with saline and the brains were sliced into 2-mm coronal slices at 24 h after tPA treatment. The slices were then photographed and homogenized in 50% trichloroacetic acid and centrifuged at 10,000 g for 30 min. The supernatants were subjected to spectrophotometric quantification of leaked Evans blue dye at an excitation wavelength of 620 nm.

For evaluation of albumin leakage, FITC-labeled albumin (50 mg/kg, Sigma-Aldrich, MO, United States) was infused via femoral vein after craniotomy at 24 h after tPA treatment. The mouse head was fixed in a stereotactic frame and the skull was thinned by an electric cranial drill. Thirty minutes after FITC infusion, the albumin leakage from cerebral venules was observed using an intravital fluorescent microscopy (BX51WI, Olympus, Tokyo, Japan). Fluorescence signal (excitation wavelength at 420–490 nm, emission wavelength at 520 nm) was acquired using a super-sensitive CCD camera (USS-301, UNIQ, CA, United States). The fluorescent intensity was measured using ImageJ software (NIH, Bethesda, MD, United States). The results were presented as I/V (I, the fluorescent intensity in interstitial tissue; V, the fluorescent intensity within cerebral venule) (Li et al., 2019).

### Monitoring of Cerebral Blood Flow

The cerebral blood flow (CBF) was determined using a laser-Doppler flowmetry (moorFLPI-2, Moor Instruments, Devon, UK). For this, a skin incision was performed to expose the skull. The whole brain scan was performed using the probe to measure CBF. Blood flow data was recorded at baseline, 2.5, 4.5, 5.5, 6.5, and 28.5 h after FeCl_3_ stimulation, respectively (Figure 1A). The changes of CBF were analyzed using moorFLPI Review software (Moor Instruments, Devon, United Kingdom).

### Measurement of Adherent Leukocytes

Adherent leukocytes in mouse cerebral venules were measured as previously reported (Wang et al., 2019). The fluorescence tracer Rhodamine 6G (1.5 mg⁄kg, Sigma-Aldrich, MO, United States) was administrated to mouse via the femoral vein 30 min before observation. Then the cerebral cortex venules were observed after craniotomy under an intravital fluorescent microscopy (BX51WI, Olympus, Tokoy, Japan) at the wavelength of 543 nm. The adherent leukocytes were identified as those that stayed attaching to the venular walls for more than 10 s. The number of adherent leukocytes was counted manually on the captured images.

### Assessment of Neurological Deficits

Neurological Deficits of mice were assessed 24 h after tPA administration. Neurological function was graded with modified neurological severity scores (mNSS) and neurological evaluation scale (NES) as previously described (Supplementary Table S2, S3) (Chen QF. et al., 2018). Analyses were performed by a blind investigator.

### Western Blot

Western Blot was performed as described previously (Chen QF. et al., 2018). Protein extracts were obtained from the right cerebral cortices. Brain tissues were lyzed by RIPA lysis buffer (100 mg/ml). To prepare protein sample, tissue homogenates were centrifuged at 12,000 rpm for 25 min and protein concentration was determined by a BCA protein assay. After quantification, protein was separated by SDS-PAGE (10 ul homogenate per lane) and transferred to a polyvinylidene difluoride membrane. Membranes were blocked with 5% skimmed milk powder for 1 h at room temperature. Antibodies used were as follows: anti-zonula occludens-1 (1:1000, ab216880, Abcam, Cambridge, United Kingdom); anti-junctional adhesion molecule-1 (1:1000, ab180821, Abcam, Cambridge, United Kingdom); anti-Occludin (1:1000, ab224526, Abcam, Cambridge, United Kingdom); anti-Claudin 5 (1:1000, 35-2500, Invitrogen, CA, United States); anti-VE Cadherin (1:1000, ab232515, Abcam, Cambridge, United Kingdom); anti-α catenin (1:1000, 2131, CST, MA, United States); anti-β catenin (1:1000, 9562, CST, MA, United States); anti- ATP 5D (1:1000, ab97491, Abcam, Cambridge, United Kingdom); anti-ATP synthase α (1:1000, 612,517, BD, NJ, United States); anti-Caveolin-1 (1:1000, 3238, CST, MA, United States); anti-Src (1:1000, 2107, CST, MA, United States); anti-p-Src (1:1000, ab32078, Abcam, Cambridge, United Kingdom); anti-Collagen IV (1:1000, ab6586, Abcam, Cambridge, United Kingdom); anti-Laminin (1:1000, ab11575, Abcam, Cambridge, United Kingdom); anti-MMP2 (1:1000, ab37150, Abcam, Cambridge, United Kingdom); anti-MMP9 (1:1000, ab228402, Abcam, Cambridge, United Kingdom); anti-β actin (1:2000, ab8226, Abcam, Cambridge, United Kingdom). Then the membranes were incubated with a secondary antibody for 1 hour at room temperature. The protein bands were detected with an enhanced chemiluminescence system and quantification analyses were performed using ImageJ software (NIH, Bethesda, MD, United States).

### Immunofluorescence Staining and Immunohistochemistry

Immunofluorescence staining was performed as described previously (Chen QF. et al., 2018). The brain frozen tissues were sectioned with a cryostat (CM 1900, Leica, Bensheim, Germany) to a thickness of 10 μm. The sections were blocked with goat serum for 15 min at room temperature. Antibodies used were as follows: anti-Occludin (1:200, ab224526, Abcam, Cambridge, United Kingdom); anti-VE cadherin (1:200, ab232515, Abcam, Cambridge, United Kingdom); anti-Collagen IV (1:500, ab6586, Abcam, Cambridge, United Kingdom); anti-Laminin (1:400, ab11575, Abcam, Cambridge, United Kingdom); anti-MMP9 (1:500, ab38898, Abcam, Cambridge, United Kingdom); anti-MPO (1:200, ab9535, Abcam, Cambridge, United Kingdom); anti-von Willebrand factor (1:50, GTX28822, Gene Tex, CA, United States); anti-GFAP (1:200, ab279289, Abcam, Cambridge, United Kingdom); anti-AQP4 (1:200, ab259318, Abcam, Cambridge, United Kingdom); anti-CD18 (1:100, ab52920, Abcam, Cambridge, United Kingdom); anti-CD68 (1:100, ab31630, Abcam, Cambridge, United Kingdom). Primary antibodies were recognized by the following secondary antibodies: DyLight 488-labeled goat anti-rabbit IgG (1:100, KPL, MD, United States) and DyLight 549-labeled goat anti-rabbit IgG (1:100, KPL, MD, United States). Hoechst 33,342 (1:50, H342, Dojindo, Kumamoto, Japan) was used to stain nuclei. Slices were photographed under a laser scanning confocal microscope (TCS SP5, Leica, Bensheim, Germany).

Immunohistochemistry was undertaken as routine. After antigen retrieval and blocking steps (sections were blocked with goat serum for 20 min at room temperature), 10 μm sections were incubated with primary antibodies as follows: anti-MMP2 (1:200, ab92536, Abcam, Cambridge, United Kingdom); anti-MMP9 (1:500, ab38898, Abcam, Cambridge, United Kingdom); anti-MPO (1:200, ab9535, Abcam, Cambridge, United Kingdom). Specific binding was detected by incubation with an HRP-conjugated secondary antibody and revealed using the DAB substrate kit (Zhong Shan-Golden Bridge Biological Technology, Beijing, China). Slices were observed and photographed under an optical microscope (BX512DP7, Olympus, Tokyo, Japan).

### Statistical Analysis

Data analysis was performed using GraphPad Prism version 7.0 software for Windows (GraphPad Software, CA, United States). Results were expressed as mean ± SEM. The Shapiro-Wilk test was performed to assess the normality of data distribution. When passing the normality test, a one-way or two-way ANOVA followed by Tukey’s post hoc test was used for statistical analysis. Otherwise, a Kruskal–Wallis test was performed. A Log-rank (Mantel-Cox) test was performed to analyze the Kaplan-Meier survival curves. *p* < 0.05 was considered statistically significant.

## Results

### QSYQ Reduces Delayed tPA-Induced Brain Impairment After Ischemic Stroke in Mice

We first evaluated the effect of QSYQ on CBF in tPA-treated stroke mice, which was recorded at baseline, 2.5, 4.5, 5.5, 6.5, and 28.5 h after stroke onset (Figures 1B,C). Compared with the sham group, CBF in the thrombus group was significantly decreased after 2.5 h. Neither QSYQ nor tPA alone had any effect on the reduced CBF at any time point examined. Interestingly, thrombus + tPA + QSYQ 0.5 group partly reversed the reduction of CBF at 28.5 h after stroke compared with the thrombus group. But other tPA + QSYQ treatment groups did not reveal significant effect on CBF. Similar results were observed using the India ink perfusion method (Supplementary Figure S3). We also investigated whether QSYQ directly inhibits the action of tPA *in vitro*. The results showed that QSYQ did not directly affect tPA’s fibrinolytic activity (Supplementary Figure S4).

We then quantified the effect of QSYQ on brain infarction in tPA-treated stroke mice. Infarct volume in the thrombus + tPA group was increased compared with the thrombus group, but the difference was not statistically significant (Figures 1D,E). The increase of infarct volume in tPA-treated stroke mice was markedly reversed by QSYQ treatment at 0.5 g/kg and 1 g/kg.

### QSYQ Improves tPA-Induced Poor Survival Rate and Neurological Deficits After Ischemic Stroke

**FIGURE 2:**
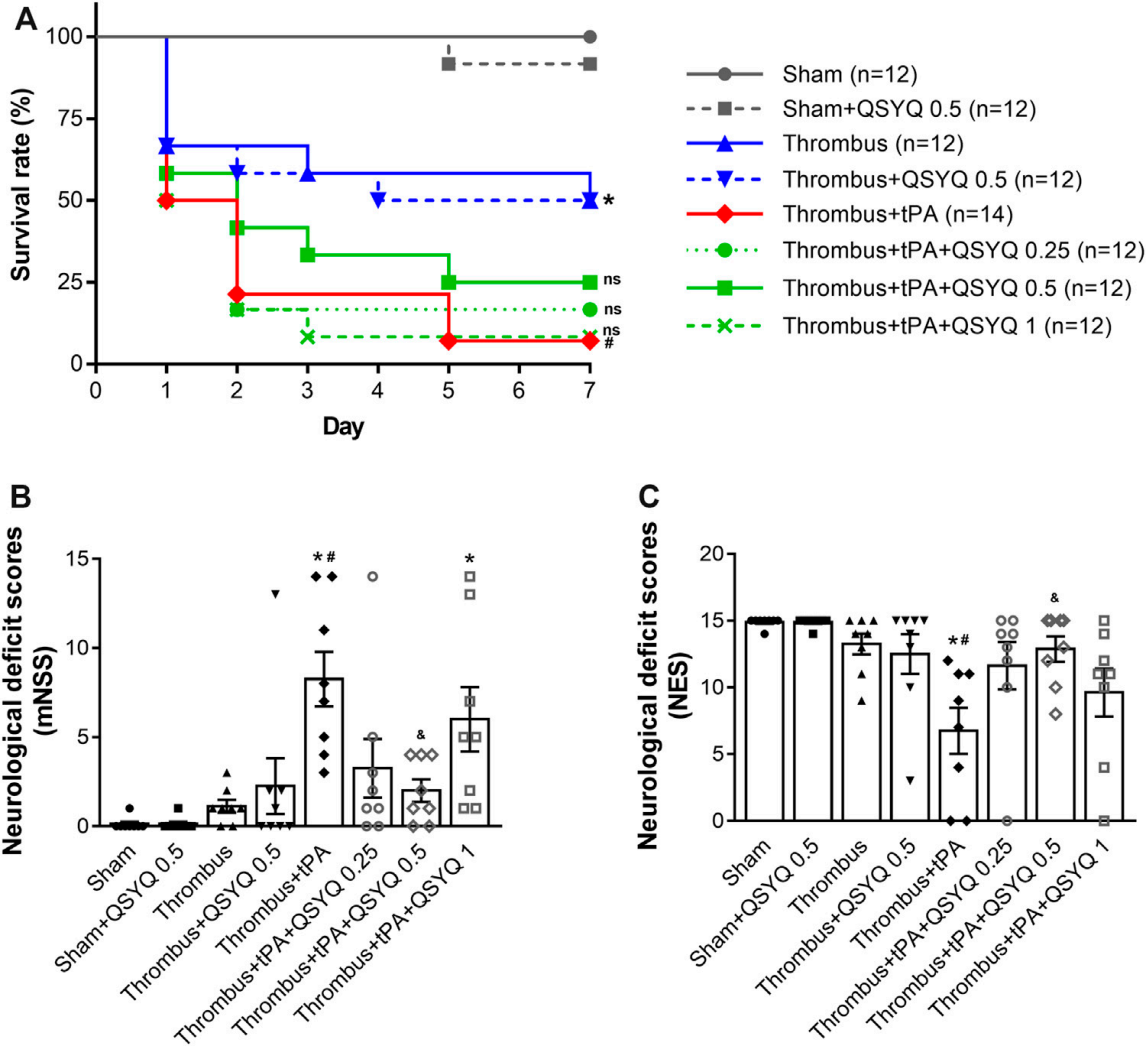
Effect of QSYQ on animal survival and neurological dysfunction in tPA-treated stroke mice. **(A)** Survival rate (n = 12 per group). Data were compared by a Log-rank (Mantel-Cox) test. Neurological deficits were assessed by **(B)** modified neurological severity score (mNSS) and **(C)** neurological evaluation scale (NES) (n = 8 per group). Data were compared by a Kruskal–Wallis test. *p < 0.05 vs sham; ^**#**^ *p* < 0.05 vs thrombus; ^&^ *p* < 0.05 vs thrombus + tPA. ns, not statistically significant compared to thrombus + tPA.

Next, we evaluated the effect of QSYQ on tPA-related poor survival rate and neurological deficits in stroke mice. The survival rate of mice was observed daily until day 7. The survival rate of thrombus group was 50% on day 7, which was significantly deteriorated by treatment with tPA (7.14%). Addition of QSYQ at 0.25 g/kg, 0.5 g/kg, 1 g/kg to tPA treatment group elevated the survival rate (16.67, 25, 8.33%, respectively; Figure 2A), although the improvement was not statistically significant.

Neurological deficits were evaluated 24 h after tPA treatment using two classic scales (mNSS and NES). Both scales showed that the neurological deficits were significantly worse in the tPA treatment group than that in the thrombus group. Interestingly, the aggravating neurological deficits in tPA-treated stroke mice were markedly reversed by addition of QSYQ at 0.5 g/kg. While addition of QSYQ at 0.25 g/kg or 1 g/kg to tPA treatment group did not reveal significant effect on neurological deficit scores (Figures 2B,C). We also observed the efficacy of QSYQ on tPA-induced neurological deficits at 2 weeks after stroke onset (Supplementary Figure S5). The results showed that neurological deficits were dramatically worse after tPA treatment. Addition of QSYQ at 0.5 g/kg attenuated the neurological deficits induced by tPA administration, although the difference was not statistically significant.

### QSYQ Prevents tPA-Induced Blood–Brain Barrier Deterioration and Brain Edema After Ischemic Stroke

**FIGURE 3:**
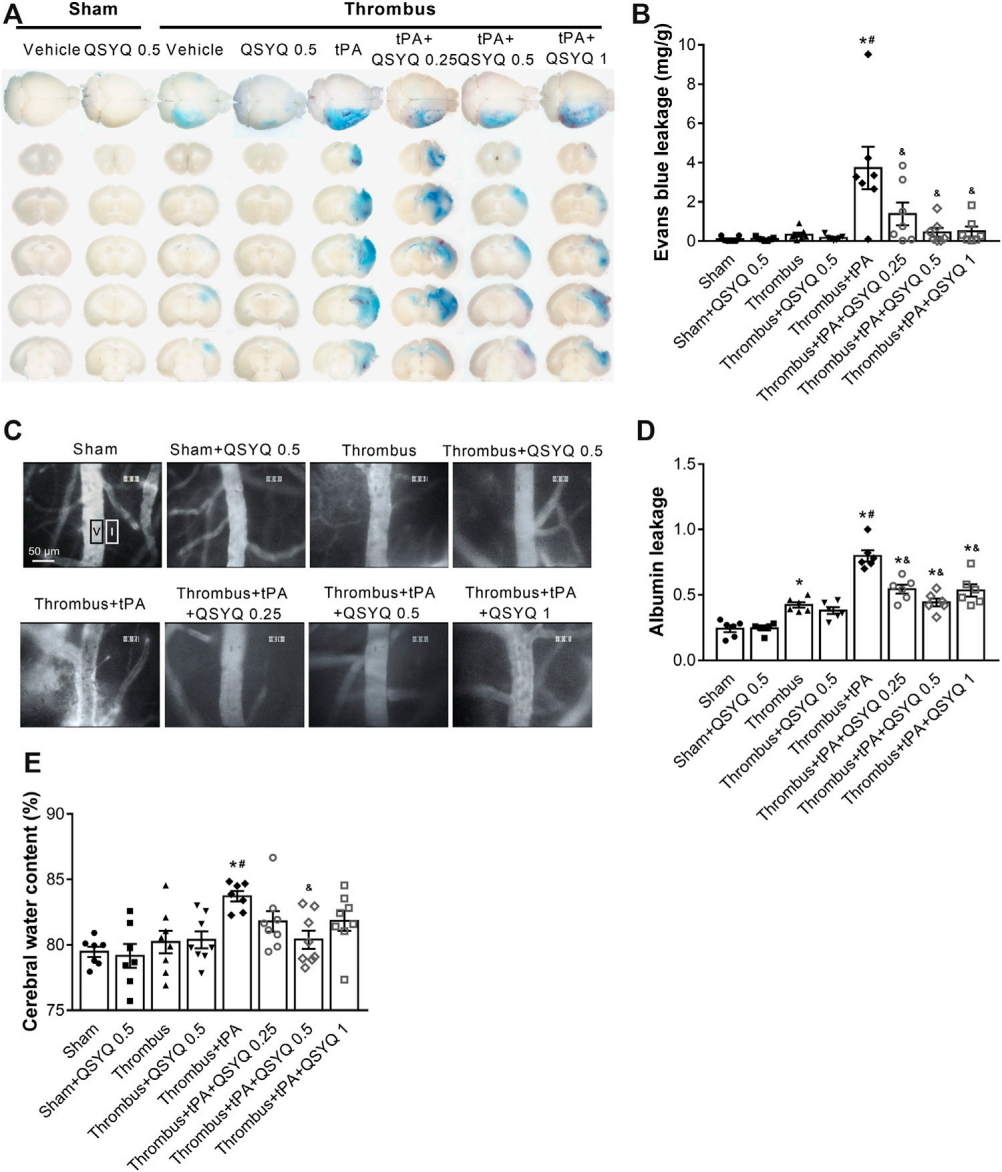
Effect of QSYQ on blood-brain barrier (BBB) permeability in tPA-treated stroke mice. **(A)** Representative images and **(B)** quantitative analysis of Evans blue extravasation (n = 7 per group). Data were compared by a two-way ANOVA followed by Tukey’s post hoc test. **(C)** Representative images and **(D)** quantitative analysis of albumin leakage (n = 6 per group). Data were compared by a two-way ANOVA followed by Tukey’s post hoc test. **(E)** Quantitative analysis of cerabral water content (n = seven to eight per group). Data were compared by a two-way ANOVA followed by Tukey’s post hoc test. *p < 0.05 vs sham; ^**#**^ *p* < 0.05 vs thrombus; ^&^ *p* < 0.05 vs thrombus + tPA.

We further explored whether QSYQ inhibits BBB disruption and brain edema in tPA-treated stroke mice. The Evans blue extravasation and albumin leakage were used to quantify BBB damage. In stroke mice, Evans blue extravasation was dramatically increased after tPA treatment (Figures 3A,B). Addition of QSYQ at 0.25 g/kg, 0.5 g/kg, and 1 g/kg significantly reduced the extravasation of Evans blue in stroke mice with tPA infusion. Similarly, leakage of albumin was markedly increased in the tPA-alone group, and the tPA-associated increase was significantly attenuated by QSYQ treatment at 0.25 g/kg, 0.5 g/kg, and 1 g/kg (Figures 3C,D). Cerebral water content was used to determine brain edema. As expected, tPA treatment elicited obvious brain edema in stroke mice (Figure 3E). The increased cerebral water content was attenuated significantly by addition of QSYQ at 0.5 g/kg.

### QSYQ Reduces the Risk of tPA-Induced Brain Hemorrhage After Ischemic Stroke

**FIGURE 4:**
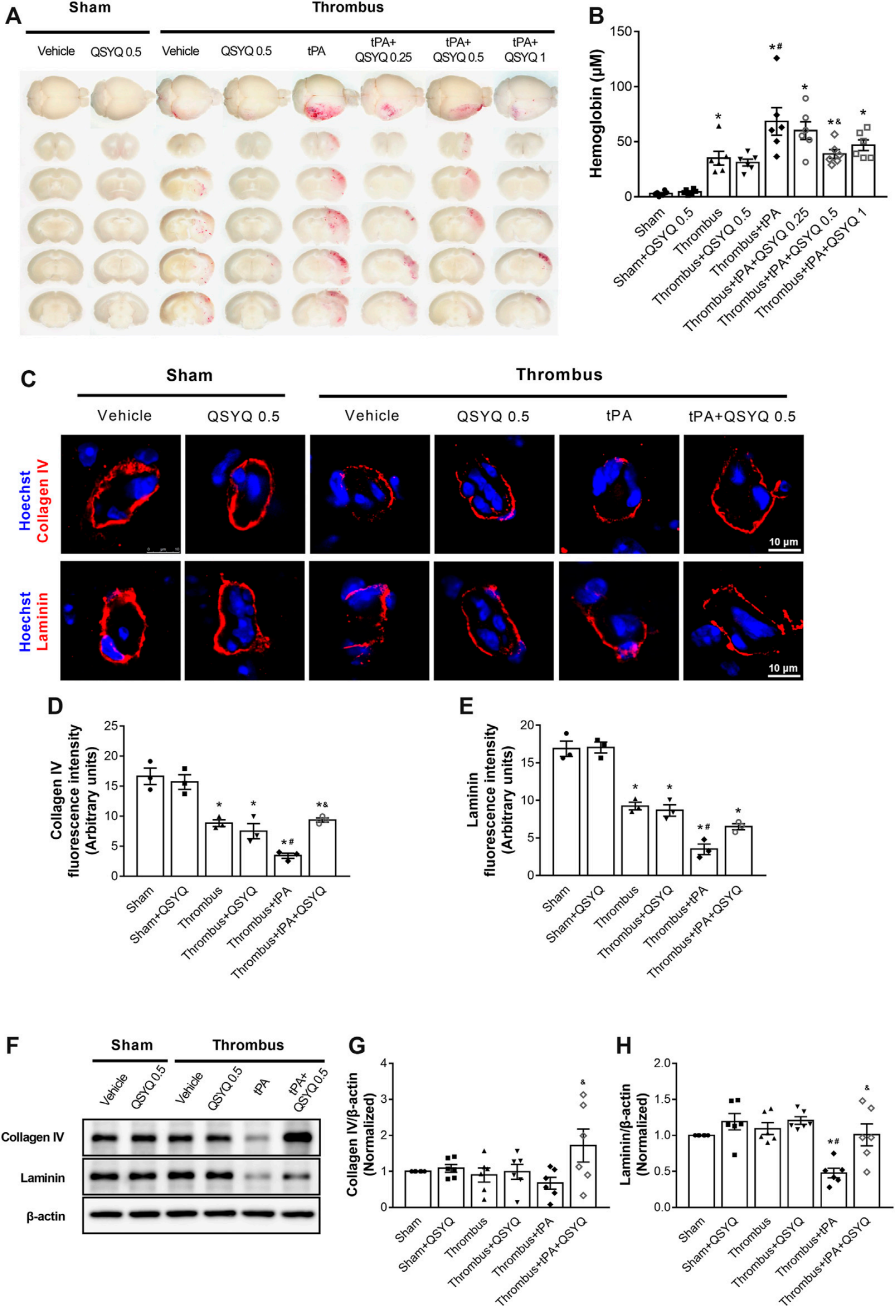
Effect of QSYQ on brain hemorrhage and basement membrane protein expression in tPA-treated stroke mice. **(A)** Representative slices and **(B)** quantitative analysis of brain hemorrhage in each group (n = 6 per group). Data were compared by a two-way ANOVA followed by Tukey’s post hoc test. **(C)** Representative images and **(D,E)** quantitative analysis of immunofluorescence staining for collagen IV and laminin in indicated groups (n = 3 per group). **(F)** Representative images and **(G,H)** quantitative analysis of immunoblotting of collagen IV and laminin in mouse brain tissues (n = 6 per group). Data were compared by a two-way ANOVA followed by Tukey’s post hoc test. *p < 0.05 vs sham; ^**#**^ *p* < 0.05 vs thrombus; ^&^ *p* < 0.05 vs thrombus + tPA.

We next evaluated the effect of QSYQ on tPA-induced brain hemorrhage in stroke mice. The degrees of hemorrhagic infarction were scored as described in the methods. The hemorrhage rate of thrombus group was 37.5%, which was increased by treatment with tPA to 70.59% with a high percentage (35.29%) of severe hemorrhage (HI-2 and HI-3). Addition of QSYQ at 0.25 g/kg, 0.5 g/kg, 1 g/kg to thrombus + tPA group decreased the hemorrhage rate (56.25, 40, 60%, respectively) with low incidence of severe hemorrhage (25, 20, 26.67%, respectively; Supplementary Figure S6). Then brain hemorrhage was quantified by the spectrophotometric hemoglobin assay, showing that infusion of tPA elicited obvious brain hemorrhage in stroke mice, which was significantly attenuated by addition of QSYQ at 0.5 g/kg (Figures 4A,B).

The expressions of collagen IV and laminin were evaluated by Western blot and immunofluorescence staining. Administration of tPA induced disruptions of collagen IV and laminin in stroke mice, while the tPA-evoked disruptions of basement membrane proteins were partly prevented by addition of QSYQ at 0.5 g/kg (Figures 4C–E). Western blot showed that the expression of laminin was significantly decreased by tPA treatment (Figures 4F–H). Addition of QSYQ at 0.5 g/kg to thrombus + tPA group markedly elevated the expression levels of collagen IV and laminin.

### QSYQ Ameliorates tPA-Induced Degradation of Tight and Adherens Junctions After Ischemic Stroke

**FIGURE 5:**
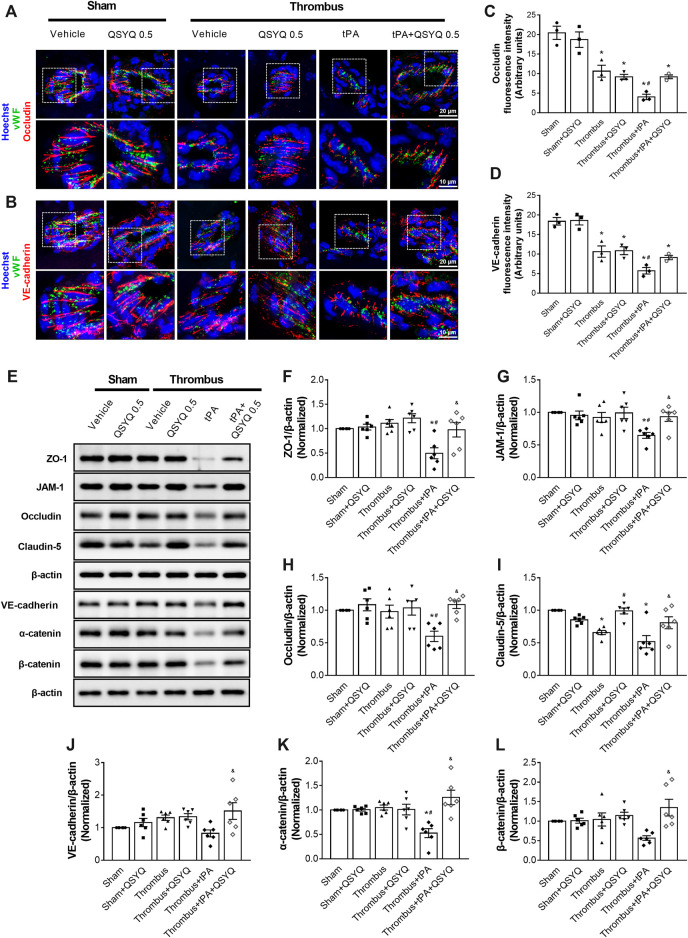
Effect of QSYQ on tight junctions and adherens junctions protein expression in tPA-treated stroke mice. **(A,B)** Representative images and **(C,D)** quantitative analysis of immunofluorescence staining for occludin and VE-cadherin in indicated groups (n = 3 per group). **(E)** Representative images of immunoblotting of zonula occludens-1 (ZO-1), junctional adhesion molecule-1 (JAM-1), occludin, claudin-5, VE-cadherin, α-catenin, and β-catenin in mouse brain tissues. Quantitative analysis of immunoblotting of ZO-1 **(F)**, JAM-1 **(G)**, occludin **(H)**, claudin-5 **(I)**, VE-cadherin **(J)**, α-catenin **(K)**, and β-catenin **(L)** (n = 6 per group). Data were compared by a two-way ANOVA followed by Tukey’s post hoc test. *p < 0.05 vs sham; ^**#**^ *p* < 0.05 vs thrombus; ^&^ *p* < 0.05 vs thrombus + tPA.

In light of the critical role of endothelial cell junction in regulation of BBB, we determined the effect of QSYQ on expressions of tight and adherens junction proteins in tPA-treated stroke mice. Immunofluorescence staining showed that tPA treatment caused discontinuity of occludin and VE-cadherin around the periphery of endothelial cells, while the discontinuity was attenuated by addition of QSYQ at 0.5 g/kg (Figures 5A–D). Western blot showed that tPA treatment induced a decreased expression of zonula occludens-1 (ZO-1), junctional adhesion molecule-1 (JAM-1), occludin, claudin-5, VE-cadherin, α-catenin, β-catenin compared with the thrombus group (Figure 5E-L). All the decreased expressions of tight and adherens junction proteins caused by tPA were protected by addition of QSYQ at 0.5 g/kg.

### QSYQ Inhibits tPA-Induced Upregulation of Src/Caveolin-1 Pathway and Attenuates Energy Metabolism Disturbance After Ischemic Stroke

**FIGURE 6:**
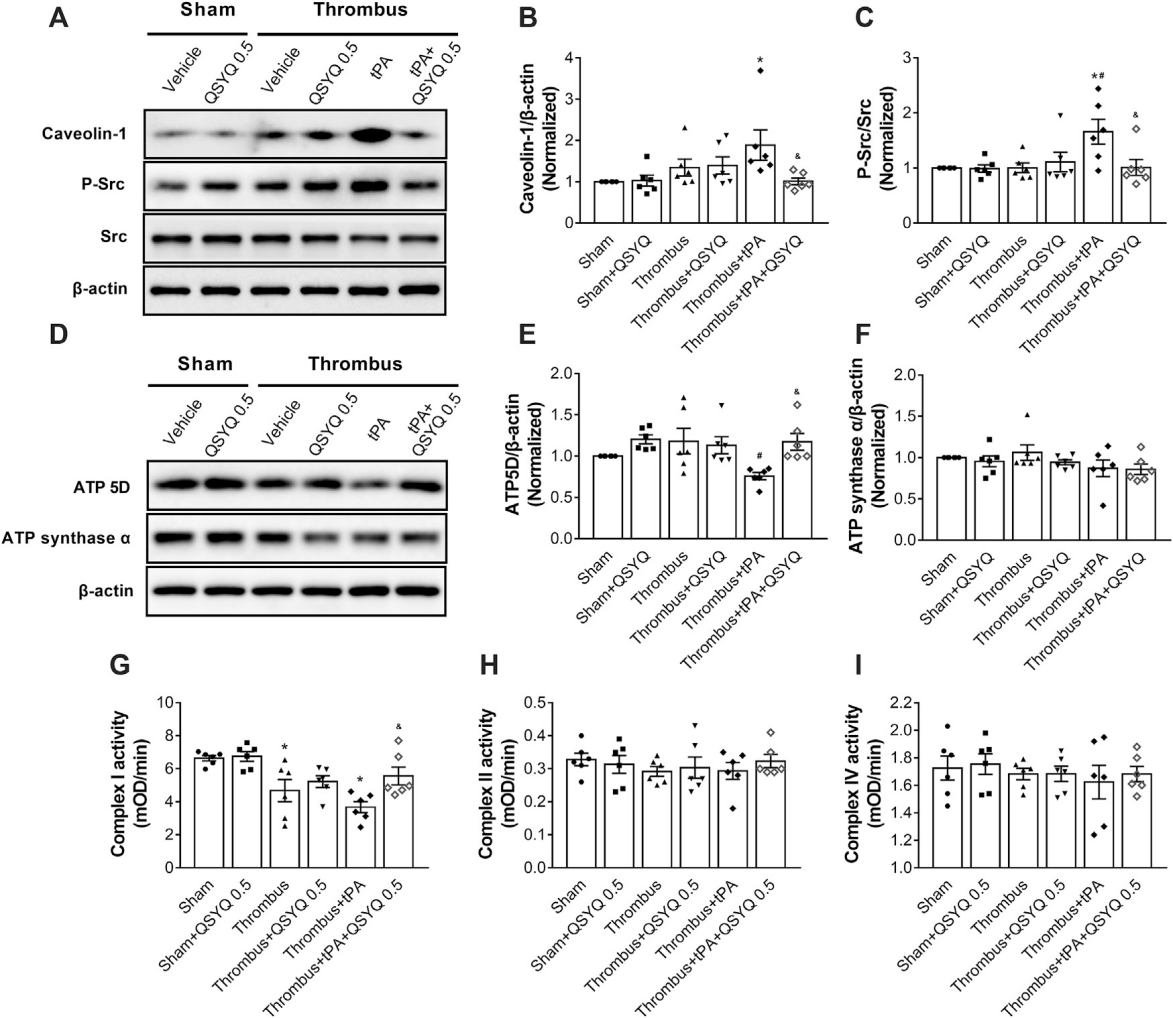
Effect of QSYQ on caveolae-related protein expression, energy-related protein expression, and mitochondrial complex enzyme activity in tPA-treated stroke mice. **(A)** Representative images and **(B,C)** quantitative analysis of immunoblotting of Caveolin-1, Src, and phospho-Src in mouse brain tissues (n = 6 per group). Data were compared by a two-way ANOVA followed by Tukey’s post hoc test. **(D)** Representative images and **(E,F)** quantitative analysis of immunoblotting of ATP 5D and ATP synthase α in mouse brain tissues (n = 6 per group). Data were compared by a two-way ANOVA followed by Tukey’s post hoc test. **(G-I)** Enzyme activities of mitochondrial Complex I, II, and IV (n = 6 per group). Data were compared by a two-way ANOVA followed by Tukey’s post hoc test. *p < 0.05 vs sham; ^**#**^ *p* < 0.05 vs thrombus; ^&^ *p* < 0.05 vs thrombus + tPA.

The expressions of Caveolin-1, Src, and Phospho-Src were determined by Western blot. Compared with the sham group, the expression of Caveolin-1 was significantly increased in the tPA-treated group (Figures 6A–C). But the increase was markedly blocked by addition of QSYQ at 0.5 g/kg. The phosphorylation level of Src was elevated by tPA treatment compared with the thrombus group, while addition of QSYQ to tPA-treated mice significantly hampered the phosphorylation of Src.

The expressions of ATP 5D and ATP synthase α were also determined. We observed that the expression of ATP 5D was decreased in tPA-infused mice compared to stroke mice (Figures 6D–F). Addition of QSYQ at 0.5 g/kg significantly prevented the decrease in the expression of ATP 5D. However, no statistically significant difference was observed in the expression of ATP synthase α between groups. The mitochondrial Complex I enzyme activity was decreased in the tPA-treated group, but addition of QSYQ at 0.5 g/kg blocked the decrease (Figure 6G). No statistically significant difference was observed in the enzyme activities of Complex II and Complex IV between groups (Figures 6H,I).

### QSYQ Inhibits tPA-Induced Activation of Macrophage-Derived MMP-9 After Ischemic Stroke

**FIGURE 7:**
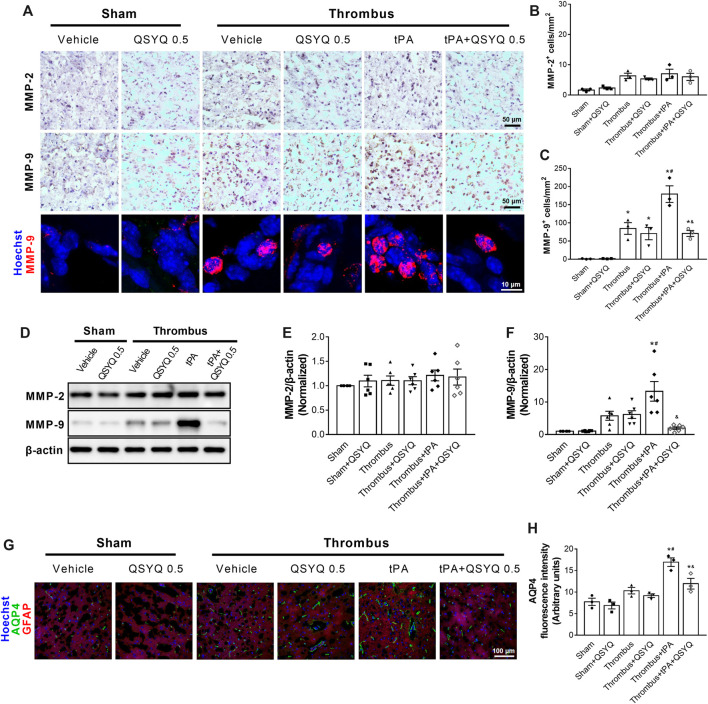
Effect of QSYQ on matrix metalloproteinases (MMPs) expression in tPA-treated stroke mice. **(A)** Representative images and **(B,C)** quantitative analysis of immunostaining results of MMP-2 and MMP-9 in indicated groups (n = 3 per group). **(D)** Representative slices and **(E,F)** quantitative analysis of immunoblotting of MMP-2 and MMP-9 in indicated groups (n = 6 per group). **(G)** Representative images and **(H)** quantitative analysis of immunofluorescence staining for aquaporin-4 (AQP4) (n = 3 per group). Data were compared by a two-way ANOVA followed by Tukey’s post hoc test. *p < 0.05 vs sham; ^**#**^ *p* < 0.05 vs thrombus; ^&^ *p* < 0.05 vs thrombus + tPA.

We sought to investigate the mechanisms by which QSYQ treatment blocked tPA-induced brain hemorrhage in stroke mice. The expressions of MMP-2 and MMP-9 were detected by immunohistochemistry staining (Figures 7A–C) and Western blot (Figures 7D–F). The results demonstrated that tPA infusion increased MMP-9 expression in the brain of stroke mice, while addition of QSYQ at 0.5 g/kg blocked the increase of MMP-9 expression. There was no obvious difference in the expression of MMP-2 among the experimental groups. We also assessed the expression of aquaporine-4 (AQP4) in this study. The results showed that the expression of AQP4 was increased in stroke mice compared to sham mice (Figures 7G,H). Administration of tPA resulted in further increase of AQP4 in stroke mice, while the tPA-evoked increase was partly prevented by addition of QSYQ at 0.5 g/kg.

**FIGURE 8:**
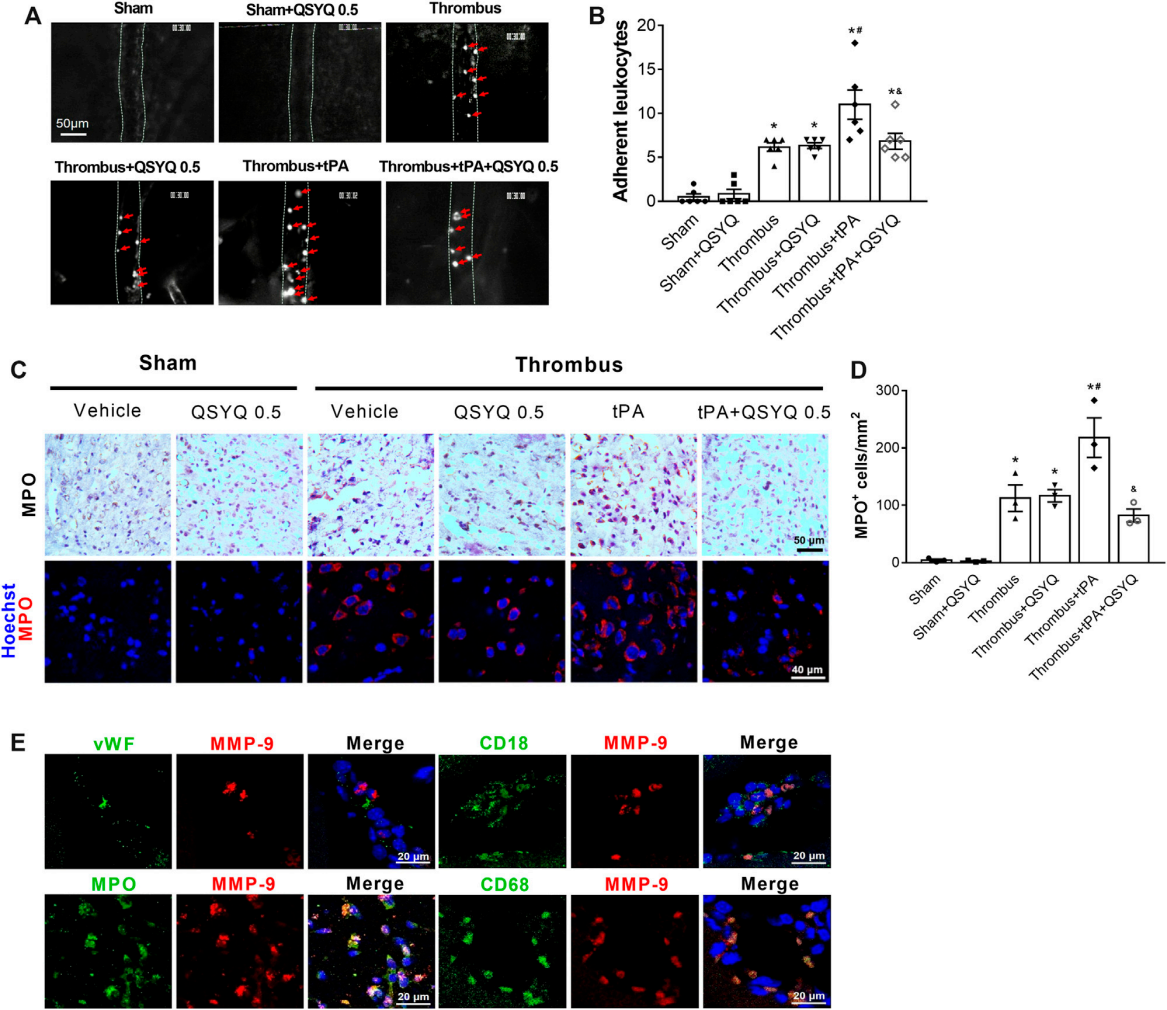
Effect of QSYQ on leukocyte adhesion and leukocyte infiltration in tPA-treated stroke mice. **(A)** Representative images and **(B)** quantitative analysis of leukocyte adhesion (n = 6 per group). Data were compared by a two-way ANOVA followed by Tukey’s post hoc test. Red arrows indicate adherent leukocytes. **(C)** Representative images and **(D)** quantitative analysis of immunostaining for myeloperoxidase (MPO) (n = 3 per group). **(E)** Double immunofluorescence staining showing the colocalization of MMP-9 with leukocyte markers (MPO, CD18) and the specific macrophage marker (CD68), but not with endothelial cell marker (von Willebrand factor, vWF) in the thrombus + tPA group (n = 3 per group). *p < 0.05 vs sham; ^**#**^ *p* < 0.05 vs thrombus; ^&^ *p* < 0.05 vs thrombus + tPA.

We assessed the effect of QSYQ on tPA-related leukocyte adhesion in cerebral venules of stroke mice. Compared with the thrombus group, the number of adherent leukocytes in the thrombus group was significantly increased after tPA infusion (Figures 8A,B). The combination therapy of QSYQ and tPA markedly attenuated the increase of adherent leukocytes. Leukocyte infiltration was quantified by myeloperoxidase (MPO) staining. MPO-positive cells were significantly increased in the brains of tPA-treated stroke mice (Figures 8C,D). Addition of QSYQ at 0.5 g/kg markedly hampered the increase of MPO-positive cells. We further explored where did the increased MMP-9 in tPA-treated mice mainly derive from (Figure 8E). Double immunostaining showed strong colocalization of MMP-9 signal with the MPO, CD18, and CD68, but not with the endothelial cell-specific marker (von Willebrand factor, vWF).

## Discussion

Delayed treatment of tPA-induced brain edema and hemorrhage in ischemic stroke limits its application in clinic. We previously reported that T541, a compound medicine consisting of major bioactive ingredients of QSYQ, attenuated brain impairment caused by delayed tPA treatment in a stroke model (Chen QF. et al., 2018). The present study, for the first time, provided direct evidence demonstrating that QSYQ attenuated tPA-induced brain edema and hemorrhage in a modified stroke model, and the effects of QSYQ were attributable to its capacity to contain BBB damage, leukocyte adhesion, leukocyte infiltration, and MMP-9 release. Moreover, the survival rate of mice was assessed daily until day 7 after initiation of stroke in present study rather than at 24 h as in study of T541 (Chen QF. et al., 2018), the result of the present study is thus more clinically relevant.

A modified FeCl_3_-induced thromboembolic stroke model (Martinez de Lizarrondo et al., 2017) was employed in this study, wherein the FeCl_3_-induced thrombi were mechanically detached allowing for movement to middle cerebral artery. Compared with the classic middle cerebral artery occlusion model (Longa et al., 1989), this model is easy to perform and well mimics the clinical process of human thromboembolic stroke. Our study showed that tPA alone treatment did not significantly restore the CBF in thrombus mice. This is probably because the thrombi induced by FeCl_3_ are platelet-rich and tPA resistant (Martinez de Lizarrondo et al., 2017). It appeared that QSYQ alone treatment had a positive effect on the CBF restoration, although the result was not statistically significant. Furthermore, the combination of tPA and QSYQ 0.5 g/kg partly reversed the CBF reduction caused by thrombus. Interestingly, QSYQ at 0.5 g/kg apparently increased the number of open cerebral vessels in mice underwent thrombus challenge, as shown in Supplementary Figure S3, which suggests QSYQ having a thrombolysis activity.

The BBB disruption is a key episode in the brain damage after delayed tPA treatment, which leads to brain edema and hemorrhage (Ye et al., 2020). BBB is maintained by endothelial cell junctions and intact basement membrane, as well as by rare transcellular transport via intracellular vesicles. Delayed tPA treatment-mediated BBB disruption in ischemic stroke is in essence an ischemia and reperfusion (I/R) injury, which superimposes on the insults caused by protease activity of tPA and impacts the BBB via a variety of mechanism. Malfunctional energy metabolism in this condition deprives ATP supply necessary for keeping F-actin intact, the junction proteins thus losses mechanic support and tends to collapse. I/R injury is known to provoke the release of MMPs resulting in degradation of junctional and basement membrane proteins, which, along with disrupted cell junctions, accounts for cerebral hemorrhage. I/R is also reported to activate Src, which initiates a spectrum of reactions including phosphorylation of caveolin-1 accelerating intracellular transport. In consistence with these reports, we observed a downregulated expression of ATP 5D in thrombus + tPA treatment group, suggesting the contribution of energy metabolism dysfunction to break down the BBB in this circumstance. Addition of QSYQ prior to tPA administration protected reduction of ATP 5D expression by tPA, an effect predicable in light of the reported role of notoginsenoside R1 (He et al., 2014), the main components of *Panax notoginseng*.

In line with other studies, our results showed that tPA evoked an increase in MMP-9 protein expression in the cortex, while no significant change was detected for MMP-2 expression (Murata et al., 2008; Jin et al., 2011). This result differs from that in T541, in which no significant change was found for the content of both MMP-2 and MMP-9 in the cortex from ischemic stroke mice after tPA treatment, although the activity of the two MMPs increased in plasma (Chen QF. et al., 2018). This inconsistence is likely due to the difference in the animal model and/or the methodology for quantifying MMPs used in the two studies. Nevertheless, the present study revealed that QSYQ prevented the increase in MMP-9 in the cortex of stroke mice after tPA treatment, suggesting that inhibition of MMP-9 expression underlies the protective effect of QSYQ on basement membrane integrity. Endothelial cells and infiltrated neutrophils are regarded as the major source of MMP-9 in the ischemic hemisphere after tPA infusion (Kasahara et al., 2012; Jin et al., 2019). In our study, the increased MMP-9 following tPA treatment was mainly derived from MPO-postive macrophages, as demonstrated by the colocalization of MMP-9 with MPO, CD68, and CD18, but not with vWF. This result indicated that inhibition of MPO-postive macrophage-derived MMP-9 was involved in the protective effect of QSYQ on tPA-induced brain edema and hemorrhage. The capacity of QSYQ to combat inflammation has been demonstrated as well by the finding in the present study that QSYQ attenuated leukocyte adhesion to and extravasation from cerebral microvessels after tPA treatment.

It is known that stroke may induce immunosuppression (Elkind et al., 2020) and tPA might aggravate this effect (Draxler et al., 2019). The present study suggested that QSYQ attenuated tPA-induced inflammation in central nerve system mainly by reducing leukocyte adhesion and suppressing leukocyte infiltration. QSYQ probably not affect the number of peripheral blood leucocytes (Han et al., 2019). Our previous studies did not observe suppressive effect of QSYQ on immune function in sham control animals (Li et al., 2012; Zhou et al., 2017), which further support our speculation.

The present study revealed that Caveolin-1, alone with p-Src, increased in ischemic stroke mouse brain tissue upon delayed treatment with tPA, consistent with the result from others (Chen S. et al., 2018), which was abrogated by addition of QSYQ, suggesting Src/Caveolin-1 as one of the targets for QSYQ to act.

The optimal dosage of QSYQ for attenuation of tPA-induced brain damage and cerebral microvascular dysfunction was explored in this study. Interestingly, we did not observe a dose-dependent effect of QSYQ on infarct volume, neurological scores, BBB damage, and hemorrhage, etc. The effect of middle dosage of QSYQ was found to be the best among the three doses tested, which suggests the importance of selecting an optimal dosage in clinic.

## Conclusion

In conclusion, this study provides the first direct evidence that QSYQ treatment may inhibit tPA delayed treatment-induced brain edema and hemorrhage after ischemic stroke. The protective effects of QSYQ are probably attributable to its ability to protect energy metabolism disorder, Caveolin-1/Src activation and inflammation, highlighting the advantage of QSYQ as a multitarget medicine. The current results support QSYQ as a promising adjunctive therapy for the treatment of brain edema and hemorrhage in acute ischemic stroke patients with tPA treatment with potential to widen the window for tPA application.

## Data Availability

The original contributions presented in the study are included in the article/Supplementary Material, further inquiries can be directed to the corresponding authors.

## References

[B1] Chen Q. F. Liu Y. Y. Pan C. S. Fan J. Y. Yan L. Hu B. H. (2018a). Angioedema and Hemorrhage after 4.5-Hour tPA (Tissue-Type Plasminogen Activator) Thrombolysis Ameliorated by T541 via Restoring Brain Microvascular Integrity. Stroke 49 (9), 2211–2219. 10.1161/STROKEAHA.118.021754 30354988

[B2] Chen S. Chen Z. Cui J. McCrary M. L. Song H. Mobashery S. (2018b). Early Abrogation of Gelatinase Activity Extends the Time Window for tPA Thrombolysis after Embolic Focal Cerebral Ischemia in Mice. eNeuro 5, e0391–0317. 10.1523/ENEURO.0391-17.2018 PMC6021166 29963617

[B3] Cheng N. T. Kim A. S. (2015). Intravenous Thrombolysis for Acute Ischemic Stroke within 3 hours Versus between 3 and 4.5 Hours of Symptom Onset. Neurohospitalist 5 (3), 101–109. 10.1177/1941874415583116 26288668 PMC4530422

[B4] De Keyser J. Gdovinová Z. Uyttenboogaart M. Vroomen P. C. Luijckx G. J. (2007). Intravenous Alteplase for Stroke: Beyond the Guidelines and in Particular Clinical Situations. Stroke 38 (9), 2612–2618. 10.1161/STROKEAHA.106.480566 17656661

[B5] Ding X. W. Sun X. Shen X. F. Lu Y. Wang J. Q. Sun Z. R. (2019). Propofol Attenuates TNF-α-Induced MMP-9 Expression in Human Cerebral Microvascular Endothelial Cells by Inhibiting Ca2+/CAMK II/ERK/NF-κB Signaling Pathway. Acta Pharmacol. Sin 40 (10), 1303–1313. 10.1038/s41401-019-0258-0 31235816 PMC6786358

[B6] Draxler D. F. Lee F. Ho H. Keragala C. B. Medcalf R. L. Niego B. (2019). t-PA Suppresses the Immune Response and Aggravates Neurological Deficit in a Murine Model of Ischemic Stroke. Front. Immunol. 10, 591. 10.3389/fimmu.2019.00591 30972077 PMC6445967

[B7] Elkind M. S. V. Boehme A. K. Smith C. J. Meisel A. Buckwalter M. S. (2020). Infection as a Stroke Risk Factor and Determinant of Outcome after Stroke. Stroke 51 (10), 3156–3168. 10.1161/STROKEAHA.120.030429 32897811 PMC7530056

[B8] García-Culebras A. Palma-Tortosa S. Moraga A. García-Yébenes I. Durán-Laforet V. Cuartero M. I. (2017). Toll-Like Receptor 4 Mediates Hemorrhagic Transformation after Delayed Tissue Plasminogen Activator Administration in *In Situ* Thromboembolic Stroke. Stroke 48 (6), 1695–1699. 10.1161/STROKEAHA.116.015956 28428349

[B9] Han J. Y. Li Q. Pan C. S. Sun K. Fan J. Y. (2019). Effects and Mechanisms of Qishenyiqi Pills and Major Ingredients on Myocardial Microcirculatory Disturbance, Cardiac Injury and Fibrosis Induced by Ischemia-Reperfusion. Pharmacol. Res. 147, 104386. 10.1016/j.phrs.2019.104386 31377222

[B10] Harada T. Kano T. Katayama Y. Matsuzaki T. Tejima E. Koshinaga M. (2005). Tissue Plasminogen Activator Extravasated Through the Cerebral Vessels: Evaluation Using a Rat Thromboembolic Stroke Model. Thromb. Haemost. 94 (4), 791–796. 10.1160/TH05-03-0164 16270632

[B11] He K. Yan L. Pan C. S. Liu Y. Y. Cui Y. C. Hu B. H. (2014). ROCK-Dependent ATP5D Modulation Contributes to the Protection of Notoginsenoside NR1 against Ischemia-Reperfusion-Induced Myocardial Injury. Am. J. Physiol. Heart Circ. Physiol. 307 (12), H1764–H1776. 10.1152/ajpheart.00259.2014 25305180

[B12] Hou S. Zhao M. M. Shen P. P. Liu X. P. Sun Y. Feng J. C. (2016). Neuroprotective Effect of Salvianolic Acids against Cerebral Ischemia/Reperfusion Injury. Int. J. Mol. Sci. 17 (7), 1190. 10.3390/ijms17071190 PMC4964559 27455249

[B13] Jin R. Song Z. Yu S. Piazza A. Nanda A. Penninger J. M. (2011). Phosphatidylinositol-3-Kinase Gamma Plays a Central Role in Blood-Brain Barrier Dysfunction in Acute Experimental Stroke. Stroke 42 (7), 2033–2044. 10.1161/STROKEAHA.110.601369 21546487 PMC3129812

[B14] Jin R. Xiao A. Y. Li J. Wang M. Li G. (2019). PI3Kγ (Phosphoinositide 3-Kinase-γ) Inhibition Attenuates Tissue-Type Plasminogen Activator-Induced Brain Hemorrhage and Improves Microvascular Patency after Embolic Stroke. Hypertension 73 (1), 206–216. 10.1161/HYPERTENSIONAHA.118.12001 30571560 PMC6309896

[B15] Kasahara Y. Nakagomi T. Matsuyama T. Stern D. Taguchi A. (2012). Cilostazol Reduces the Risk of Hemorrhagic Infarction after Administration of Tissue-Type Plasminogen Activator in a Murine Stroke Model. Stroke 43 (2), 499–506. 10.1161/STROKEAHA.111.635417 22033992

[B16] Knecht T. Story J. Liu J. Davis W. Borlongan C. V. Dela Peña I. C. (2017). Adjunctive Therapy Approaches for Ischemic Stroke: Innovations to Expand Time Window of Treatment. Int. J. Mol. Sci. 18 (12), 2756. 10.3390/ijms18122756 PMC5751355 29257093

[B17] Li D. T. Sun K. Huang P. Pan C. S. Yan L. Ayan A. (2019). Yiqifumai Injection and its Main Ingredients Attenuate Lipopolysaccharide-Induced Cerebrovascular Hyperpermeability through a Multi-Pathway Mode. Microcirculation 26 (7), e12553. 10.1111/micc.12553 31059171

[B18] Li Y. C. Liu Y. Y. Hu B. H. Chang X. Fan J. Y. Sun K. (2012). Attenuating Effect of Post-Treatment with QiShen YiQi Pills on Myocardial Fibrosis in Rat Cardiac Hypertrophy. Clin. Hemorheol. Microcirc. 51 (3), 177–191. 10.3233/CH-2011-1523 22240383

[B19] Longa E. Z. Weinstein P. R. Carlson S. Cummins R. (1989). Reversible Middle Cerebral Artery Occlusion without Craniectomy in Rats. Stroke 20 (1), 84–91. 10.1161/01.str.20.1.84 2643202

[B20] Mao L. Li P. Zhu W. Cai W. Liu Z. Wang Y. (2017). Regulatory T Cells Ameliorate Tissue Plasminogen Activator-Induced Brain Haemorrhage after Stroke. Brain 140 (7), 1914–1931. 10.1093/brain/awx111 28535201 PMC6059175

[B21] Martinez de Lizarrondo S. Gakuba C. Herbig B. A. Repessé Y. Ali C. Denis C. V. (2017). Potent Thrombolytic Effect of N-Acetylcysteine on Arterial Thrombi. Circulation 136 (7), 646–660. 10.1161/CIRCULATIONAHA.117.027290 28487393 PMC5560034

[B22] Murata Y. Rosell A. Scannevin R. H. Rhodes K. J. Wang X. Lo E. H. (2008). Extension of the Thrombolytic Time Window with Minocycline in Experimental Stroke. Stroke 39 (12), 3372–3377. 10.1161/STROKEAHA.108.514026 18927459 PMC3705574

[B23] Niego B. Medcalf R. L. (2014). Plasmin-Dependent Modulation of the Blood-Brain Barrier: A Major Consideration During tPA-Induced Thrombolysis? J. Cereb. Blood Flow Metab. 34 (8), 1283–1296. 10.1038/jcbfm.2014.99 24896566 PMC4126105

[B24] Shang H. Zhang J. Yao C. Liu B. Gao X. Ren M. (2013). Qi-Shen-Yi-Qi Dripping Pills for the Secondary Prevention of Myocardial Infarction: A Randomised Clinical Trial. Evid. Based Complement. Alternat Med. 2013, 738391. 10.1155/2013/738391 23935677 PMC3725842

[B25] Shao Y. Zhang W. Tong L. Huang J. Li D. Nie W. (2017). Simultaneous Determination of Eight Bioactive Components of Qishen Yiqi Dripping Pills in Rat Plasma Using UFLC-MS/MS and its Application to A Pharmacokinetic Study. Biomed. Chromatogr. 31 (8), e3941. 10.1002/bmc.3941 28146302

[B26] Wang H. L. Zhou Q. H. Xu M. B. Zhou X. L. Zheng G. Q. (2017). Astragaloside IV for Experimental Focal Cerebral Ischemia: Preclinical Evidence and Possible Mechanisms. Oxid Med. Cel Longev 2017, 8424326. 10.1155/2017/8424326 PMC5337886 28303172

[B27] Wang H. M. Huang P. Li Q. Yan L. L. Sun K. Yan L. (2019). Post-Treatment with Qing-Ying-Tang, a Compound Chinese Medicine Relives Lipopolysaccharide-Induced Cerebral Microcirculation Disturbance in Mice. Front. Physiol. 10, 1320. 10.3389/fphys.2019.01320 31708795 PMC6823551

[B28] Wang Y. He S. Liu X. Li Z. Zhu L. Xiao G. (2021). Galectin-3 Mediated Inflammatory Response Contributes to Neurological Recovery by QiShenYiQi in Subacute Stroke Model. Front. Pharmacol. 12, 588587. 10.3389/fphar.2021.588587 33953667 PMC8089377

[B29] Wang Y. Xiao G. He S. Liu X. Zhu L. Yang X. (2020). Protection against Acute Cerebral Ischemia/Reperfusion Injury by Qishenyiqi Via Neuroinflammatory Network Mobilization. Biomed. Pharmacother. 125, 109945. 10.1016/j.biopha.2020.109945 32028240

[B30] Xie W. Zhou P. Sun Y. Meng X. Dai Z. Sun G. (2018). Protective Effects and Target Network Analysis of Ginsenoside Rg1 in Cerebral Ischemia and Reperfusion Injury: A Comprehensive Overview of Experimental Studies. Cells 7 (12), 270. 10.3390/cells7120270 PMC6316103 30545139

[B31] Ye Y. Zhu Y. T. Tong H. X. Han J. Y. (2020). The Protective Role of Immunomodulators on Tissue-Type Plasminogen Activator-Induced Hemorrhagic Transformation in Experimental Stroke: A Systematic Review and Meta-Analysis. Front. Pharmacol. 11, 615166. 10.3389/fphar.2020.615166 33424615 PMC7793743

[B32] Zhang L. Xu S. Wu X. Chen J. Guo X. Cao Y. (2020). Combined Treatment with 2-(2-Benzofu-Ranyl)-2-Imidazoline and Recombinant Tissue Plasminogen Activator Protects Blood-Brain Barrier Integrity in a Rat Model of Embolic Middle Cerebral Artery Occlusion. Front. Pharmacol. 11, 801. 10.3389/fphar.2020.00801 32595494 PMC7303334

[B33] Zhao Z. Nelson A. R. Betsholtz C. Zlokovic B. V. (2015). Establishment and Dysfunction of the Blood-Brain Barrier. Cell 163 (5), 1064–1078. 10.1016/j.cell.2015.10.067 26590417 PMC4655822

[B34] Zheng Q. N. Wei X. H. Pan C. S. Li Q. Liu Y. Y. Fan J. Y. (2019). QiShenYiQi Pills® Ameliorates Ischemia/Reperfusion-Induced Myocardial Fibrosis Involving RP S19-Mediated TGFβ1/Smads Signaling Pathway. Pharmacol. Res. 146, 104272. 10.1016/j.phrs.2019.104272 31085230

[B35] Zhou L. Wei X. H. Pan C. S. Yan L. Gu Y. Y. Sun K. (2017). QiShenYiQi Pills, a Compound Chinese Medicine, Prevented Cisplatin Induced Acute Kidney Injury via Regulating Mitochondrial Function. Front. Physiol. 8, 1090. 10.3389/fphys.2017.01090 29312001 PMC5743021

