# Supplementary material for: QiShenYiQi Inhibits Tissue Plasminogen Activator–Induced Brain Edema and Hemorrhage after Ischemic Stroke in Mice
Source: Front Pharmacol. 2022 Jan 12;12:759027. doi: 10.3389/fphar.2021.759027 (PMC8790519; doi:10.3389/fphar.2021.759027)
Supplement: Supplementary file 1 [file DataSheet1.PDF]

Supplementary Material

1 Supplementary Methods

1.1 Indian Ink Perfusions

Mice were anesthetized and transcardially perfused with heparinized 0.9% saline to flush out the blood 24 h after tPA administration. Then the Indian ink (25%, Phygene, Fuzhou, China) made in 6% gelatin and saline was injected. After perfusion the head was cooled (4°C) for 2 hours to allow gelatin solidification. The brain was removed and was sliced into five serial 2-mm-thick coronal sections. Each section was photographed using a digital camera.

1.2 Nissl Staining

The sections were stained with cresyl violet according to the standard protocol. All images were captured under a light microscope. Adjustment of white balance was applied to the entire images.

1.3 Detection of Enzyme Activity

The mitochondrial Complex I (ab109721, Abcam, Cambridge, UK), Complex II (ab109908, Abcam, Cambridge, UK), and Complex IV (ab109911, Abcam, Cambridge, UK) enzyme activities were detected by enzyme activity microplate assay kits according to the manufacturer's instructions.

1.4 Detection of tPA Activity

The tPA activity was assessed by enzyme activity microplate assay kits according to the manufacturer's instructions (ab108905, Abcam, Cambridge, UK). Briefly, the desired volume of Assay Mix (including 60 µl Diluent, 10 µl Plasminogen, and 10 µl Plasmin Substrate) were prepared as instructed. Then 20 µl of tPA Standard and QSYQ (or vehicle) were added to each well and mix gently. The absorbance was read every hour for up to 8 hours at wavelength 450 nm.

2 Supplementary Figures and Tables

2.1 Supplementary Figures

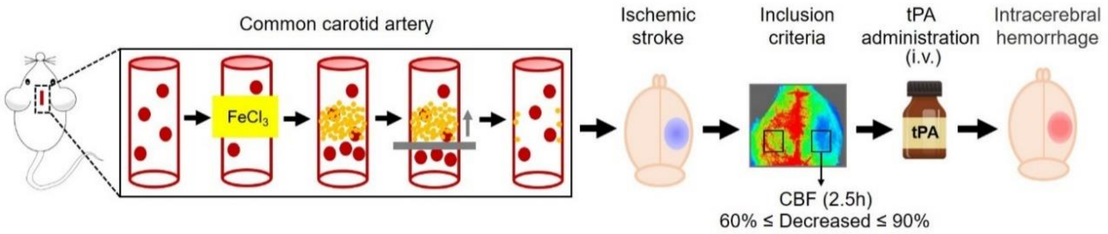

Supplementary Figure S1. Schematic representation of the different steps of tissue plasminogen activator (tPA)-induced hemorrhage model. CBF indicates cerebral blood flow.

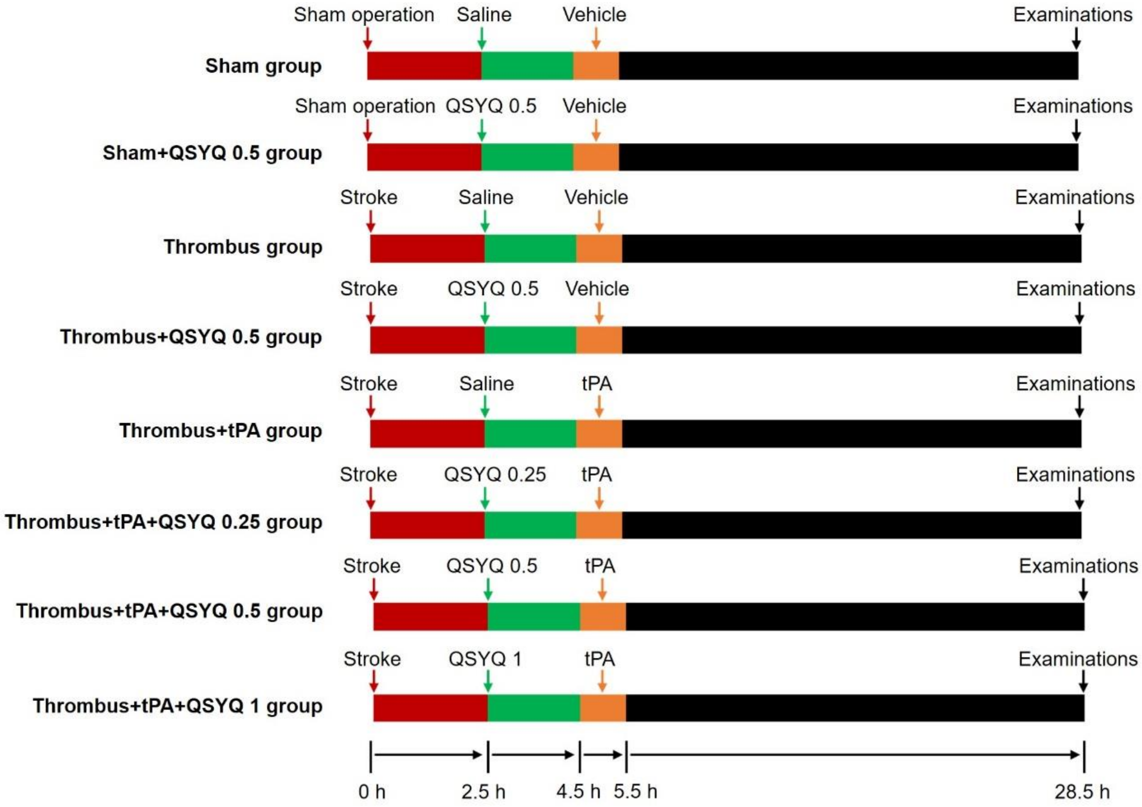

Supplementary Figure S2. The flow chart of experiments for each group.

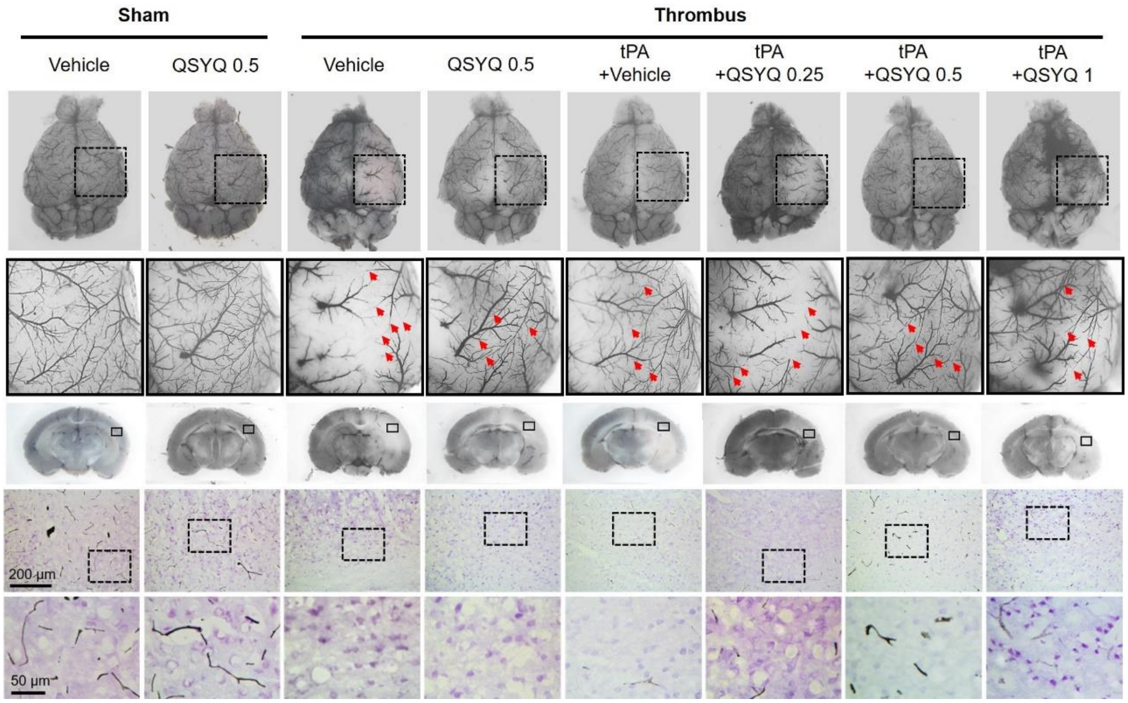

Supplementary Figure S3. Effect of QiShenYiQi Pills (QSYQ) on cerebrovascular density in tissue plasminogen activator (tPA)-treated stroke mice. The first three rows: angiograms with gelatin-India ink solution. Red arrows indicate blocked blood vessels. The last two rows: Nissl staining.

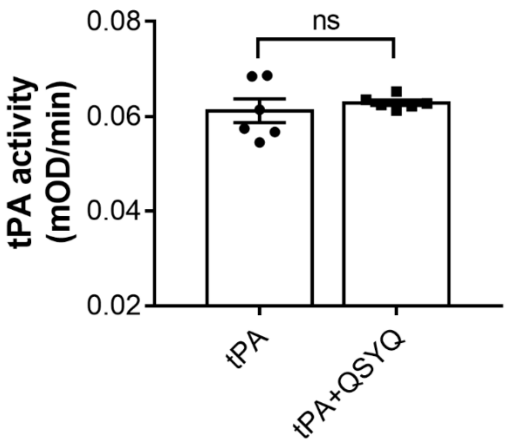

Supplementary Figure S4. QSYQ did not affect tPA's fibrinolytic function. t-PA fibrinolytic activity measured in the presence or absence of 20 μl QSYQ (0.1 g/ml). n=6 per group. Data were compared by a student's t test. ns, not statistically significant.

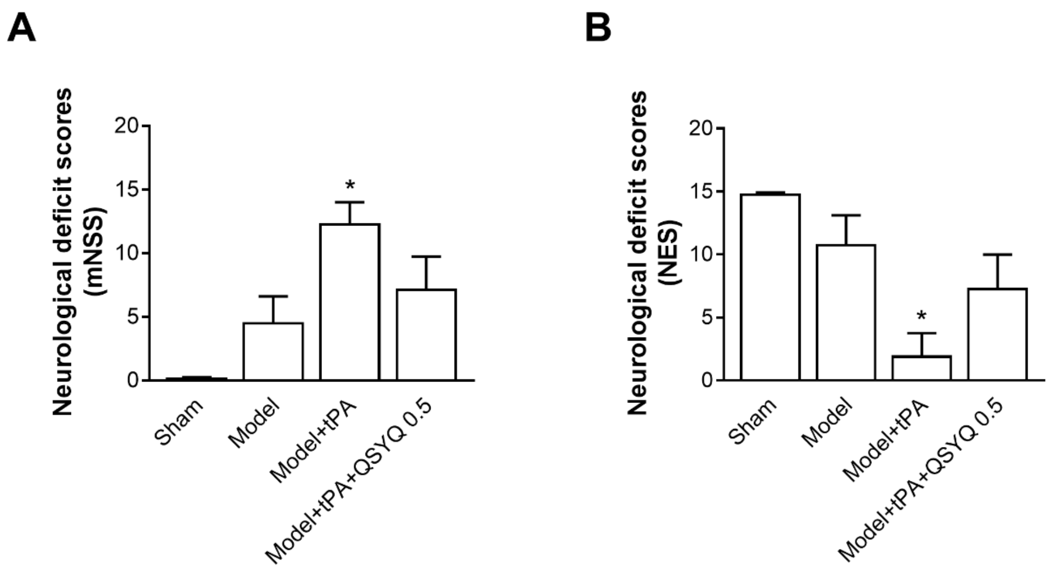

Supplementary Figure S5. Effect of QSYQ on neurological dysfunction at 2 weeks after stroke onset. Neurological deficits were assessed by **(A)** modified neurological severity score (mNSS) and **(B)** neurological evaluation scale (NES) (n=8 per group). Data were compared by a Kruskal–Wallis test. *P<0.05 vs sham.

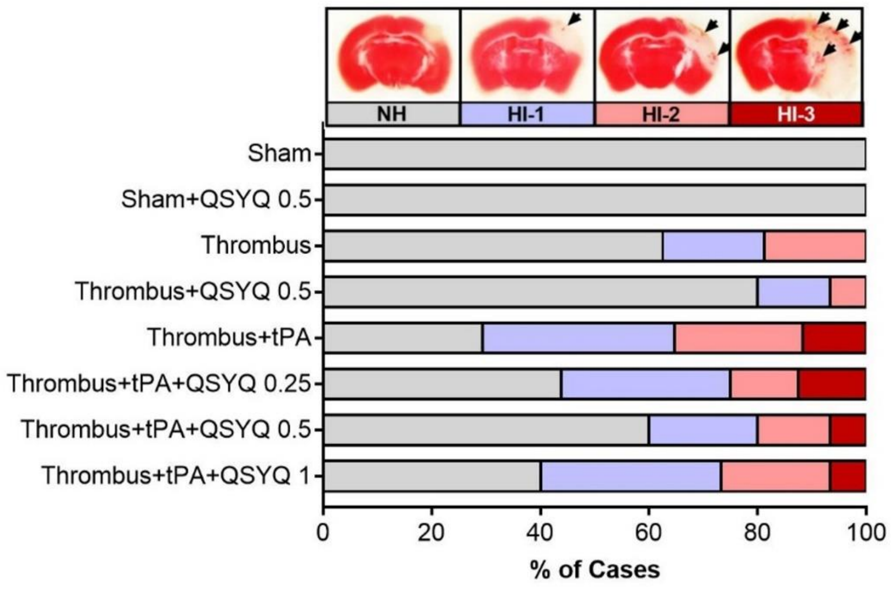

Supplementary Figure S6. Distribution of each hemorrhagic severity in each group.

2.2 Supplementary Tables

Supplementary Table S1. Animal numbers for each experiment in each group.

Group	mNSS &NES (TTC)	Suvival rate	Water content (CBF)	Evans blue	Hemoglobin	Albumin leakage (Leukocyte adhesion)	Western blot	IHC	Total
Sham+vehicle	8 (8)	12	7 (7)	7	6	6 (6)	6	3	55
Sham+QSYQ	8 (8)	12	7 (6)	7	6	6 (6)	6	3	55
Thrombus+vehicle	8 (8)	12	8 (7)	7	6	6 (6)	6	3	56
Thrombus+QSYQ	8 (8)	12	8 (6)	7	6	6 (6)	6	3	56
Thrombus+tPA	8 (8)	14	7 (7)	7	6	6 (6)	6	3	57
Thrombus+tPA +QSYQ 0.25	8 (8)	12	8 (6)	7	6	6 (-)	-	-	47
Thrombus+tPA +QSYQ 0.5	8 (8)	12	8 (6)	7	6	6 (6)	6	3	56
Thrombus+tPA +QSYQ 1	8 (8)	12	8 (6)	7	6	6 (-)	-	-	47
**Total**	**64 (64)**	**98**	**61 (51)**	**56**	**48**	**48 (36)**	**36**	**18**	**429**

*A total of 192 mice were excluded.

Number in parentheses means the same animals were used for detection of neurological scores and TTC staining. The same animals were used for assessment of cerebral water content and CBF. The same animals were used for detection of albumin leakage and leukocyte adhesion. mNSS indicates modified neurological severity scores; NES, neurological evaluation scale; CBF, cerebral blood flow; IHC, immunohistochemistry; QSYQ, QiShenYiQi Pills; tPA, tissue plasminogen activator.

Supplementary Table S2. Modified neurologic severity score for mouse (mNSS).

Test Items	Score
**1. Motor tests (muscle status-hemiplegia) (normal=0; maximum=6)**	0-6
1.1. Raising the mouse by the tail: (normal=0; maximum=3)	
-Flexion of forelimb	1
-Flexion of hindlimb	1
-Head moving more than 10 degrees to the vertical axis within 30 seconds	1
1.2. Placing the mouse on the floor: (normal=0; maximum=3)	
-Normal walk	0
-Inability to walk straight	1
-Circling toward the paretic side	2
-Falling down the paretic side	3
**2. Beam balance tests (normal=0; maximum=6)**	0-6
-Balances with steady posture	0
-Grasps side of beam	1
-Hugs the beam and one limb falls down from the beam	2
-Hugs the beam and two limbs fall down, or spins on beam (>30 s)	3
-Attempts to balance on the beam but falls off (>20 s)	4
-Attempts to balance on the beam but falls off (>10 s)	5
-Falls off: no attempt to balance or hang on to the beam (<10 s)	6
**3. Reflexes absent (normal=0; maximum=2)**	0-2
-Pinna reflex (a head shake when touching the auditory meatus)	1
-Corneal reflex (an eye blink when touching the cornea with cotton)	1

Supplementary Table S3. Neurological evaluation scale (NES).

Test Items	Score
**1. Spontaneous activity over a 3-min period (normal=3; maximum=3)**	0-3
-No movement	0
-Barely moves	1
-Moves but does not approach at least three sides of cage	2
-Moves and approaches at least three sides of cage	3
**2. Symmetry of movement (raising the mouse by the tail for 30 seconds) (normal=0; maximum=3)**	0-3
-Complete asymmetry (head moving to one side)	0
-Almost asymmetry (head moving to one side, barely to another)	1
-Medium asymmetry (head moving to one side a bit more)	2
- Almost symmetry (head moving to each side almost same times)	3
**3. Open-field path linearity (normal=0; maximum=3)**	0-3
-No movement or fall on one side	0
-Circling	1
-Move amesiality	2
-Linear motion	3
**4. Walking on a 3 cm×1 cm beam (normal=3; maximum=3)**	0-3
-Fails to climb	0
-Hangs on the beam (>30 s)	1
-Stands but barely moves	2
-Normal climbing	3
**5. Response to vibrissae touch (normal=3; maximum=3)**	1-3
-No response on left side	1
-Weak response on left side	2
-Symmetrical response	3
